# Functional characterization of *Vibrio alginolyticus* T3SS regulator ExsA and evaluation of its mutant as a live attenuated vaccine candidate in zebrafish (*Danio rerio*) model

**DOI:** 10.3389/fvets.2022.938822

**Published:** 2022-08-08

**Authors:** Weijie Zhang, Liangchuan Chen, Haiyun Feng, Junlin Wang, Fuyuan Zeng, Xing Xiao, Jichang Jian, Na Wang, Huanying Pang

**Affiliations:** ^1^Fisheries College, Guangdong Ocean University, Zhanjiang, China; ^2^Guangdong Provincial Key Laboratory of Aquatic Animal Disease Control and Healthy Culture & Key Laboratory of Control for Diseases of Aquatic Economic Animals of Guangdong Higher Education Institutes, Zhanjiang, China; ^3^Chinese Academy of Inspection and Quarantine, Beijing, China

**Keywords:** type III secretory system, characteristics, live attenuated vaccine, *Vibrio alginolyticus*, *exsA* gene

## Abstract

*Vibrio alginolyticus*, a Gram-negative bacterium, is an opportunistic pathogen of both marine animals and humans, resulting in significant losses in the aquaculture industry. Type III secretion system (T3SS) is a crucial virulence mechanism of *V. alginolyticus*. In this study, the T3SS regulatory gene *exsA*, which was cloned from *V. alginolyticus* wild-type strain HY9901, is 861 bp encoding a protein of 286 amino acids. The Δ*exsA* was constructed by homologous recombination and Overlap-PCR. Although there was no difference in growth between HY9901 and Δ*exsA*, the Δ*exsA* exhibited significantly decreased extracellular protease activity and biofilm formation. Besides, the Δ*exsA* showed a weakened swarming phenotype and an ~100-fold decrease in virulence to zebrafish. Antibiotic susceptibility testing showed the HY9901Δ*exsA* was more sensitive to kanamycin, minocycline, tetracycline, gentamicin, doxycycline and neomycin. Compared to HY9901 there were 541 up-regulated genes and 663 down-regulated genes in Δ*exsA*, screened by transcriptome sequencing. qRT-PCR and β-galactosidase reporter assays were used to analyze the transcription levels of *hop* gene revealing that *exsA* gene could facilitate the expression of *hop* gene. Finally, *Danio rerio*, vaccinated with Δ*exsA* through intramuscular injection, induced a relative percent survival (RPS) value of 66.7% after challenging with HY9901 wild type strain. qRT-PCR assays showed that vaccination with Δ*exsA* increased the expression of immune-related genes, including *GATA-1, IL6, IgM*, and *TNF-*α in zebrafish. In summary, these results demonstrate the importance of *exsA* in *V. alginolyticus* and provide a basis for further investigations into the virulence and infection mechanism.

## Introduction

*Vibrio alginolyticus*, which is a gram-negative halophilic bacterium, is diffusely distributed in nature occurring in both fresh and saltwater (1, 2). Obviously, Vibriosis disease outbreaks cause significant setbacks to aquaculture. *V. alginolyticus* was considered a serious pathogenic bacterium for many marine vertebrates, including cultured fish species such as turbot, *Larimichthys crocea*, grouper, as well as invertebrates, such as shrimp and shellfish (3, 4). It is also a zoonotic bacterium that can cause food poisoning in humans, including diarrheal and septicemia (5). Antibiotic treatment is the traditional measure used to treat *V. alginolyticus* infections, and this is considered a serious concern for the escalation of antibiotic resistant Vibrios (6). Hence, it is imperative to achieve a better understanding of the pathogenic mechanisms of *V. alginolyticus* to facilitate the discovery of more effective therapies.

Type III secretion system (T3SS) plays an important role in pathogenic process, and its structural components are highly conserved among bacterial species (7–9). ExsA, as is a member of the AraC/XylS family of DNA-binding proteins, is the main transcriptional activator of T3SS gene expression (10). And A previous study showed that the expression of ~40 gene products constituting the T3SS was regulated by 10 ExsA-dependent promoters (11). Qu et al. (12) showed that deletion of *exsA* suppressed the production of T3SS pilus (PopB/D) and effector protein (ExoT/U) in *Pseudomonas aeruginosa*. Moreover, T3SS1 expression in *V. parahaemolyticus* is genetically regulated by the ExsACDE cascade in which the master transcription factor ExsA positively regulates T3SS1 expression (13, 14). Zhou speculated that ExsA could act directly on the T3SS1 promoter sequence in view of the result that ExsA can bind the upstream region of VP1668 and VP1687 (15). To summarize, the ExsA protein exhibits significant functions during many Gram-negative bacteria pathogenesis.

When *V. alginolyticus* infects the host cell, it secretes effector proteins through T3SS, thus subverting host cellular functions and resulting in cell death (16). HopPmaJ, as a type III system effector (T3SE), is considered an important virulence factor in *V. alginolyticus*. Pang et al. (17) found HopPmaJ encoded by *hop* gene in *V. alginolyticus* is pathogenic to the orange-spotted grouper (*Epinephelus coioides*), which exhibits symptoms of ulceration, hemorrhage of liver and kidney, and swelling. Furthermore, a later study revealed that regulatory protein TyeA could up-regulated the expression of HopPmaj (18). While the T3SS is highly conserved, the regulators are unique proteins with very specialized functions critical to virulence. It was reported that T3SS of *V. alginolyticus* is similar to T3SS1 of *Vibrio* parahaemolyticus in genome analysis (19), but the regulator ExsA in *V. alginolyticus* T3SS remains poorly understood.

To better understand the function of ExsA in the *V. alginolyticus* T3SS, we constructed a HY9901Δ*exsA* mutant strain, and then studied the physiology and pathogenicity of HY9901Δ*exsA*. Besides, the differentially expressed genes between HY9901 and HY9901Δ*exsA* were investigated by transcriptome sequencing. Furthermore, we have evaluated the protective efficacy of Δ*exsA*, and found that the Δ*exsA* mutant could be used as a live attenuated vaccine to combat *V. alginolyticus* in zebrafish.

## Materials and methods

### Bacterial strains, experimental fish and plasmid

In this study, *V. alginolyticus* HY9901 was isolated from diseased red snapper (*Lutjanus sanguineus*) (20). *E. coli* S17-1 (λ Pir) and suicide plasmid pDM4 were kept in our laboratory (Table 1).

**Table 1:** Bacterial strains and plasmids used in this study.

**Strains and primers**	**Relevant characteristics**	**Source**
*V.alginolyticus* HY9901	Isolated from diseased red snapper (*Lutjanus sanguineus*)	(20)
Δ*exsA*	*exsA* deletion mutant; Apr	This study
E. coli DH5α	supE44 1lacU169 (ϕ80 lacZDM15) hsdR17 recA1 gyrA96 thi-1 relA1	TakaRa
S17-1λpir	T prSmrrecA thi pro hsdR-M+RP4:2-Tc: Mu: K m T n7λpir	(18)
pDM4	A suicide vector with ori R6K sacB; Cmr	(21)
pDM4_exsA_A1F +A2R	Flanking region sequences of exsA cloned into pDM4	This study
pME6522-*hop–lacZ*	Promoter sequences of hop cloned into pME6522	This study
S17-*hop*–lacZ	S17 carrying pME6522-hop–lacZ	This study
*hop*–lacZ: Δ*exsA*	Promoter sequences of hop was inserted in the upstream of lacZ gene in Δ*exsA* strain	This study
*hop*–lacZ: HY9901	Promoter sequences of hop was inserted in the upstream of lacZ gene in HY9901 strain	This study

### Experimental fish

Healthy zebrafish (*Danio rerio*) were purchased from a commercial fish farm in Guangzhou, China, 3–4 cm long and 0.3 g in weight. The zebrafish were tested by the bacterial recovery assay, kept in freshwater in the circulatory system at 28°C for 2 weeks before the experiment.

### Reagents and primers

TIANamp Bacteria DNAKit (Beijing, Tiangen Biotech Co., Ltd.); Easy PureTMQuick GelExtraction Kit and Easy PureTMPlasmid MiniPrepKit (Beijing, TransGen Biotech Co., Ltd.); pDM4 vector, ExTaqDNA polymerase, Prime START MHS DNA polymerase, BglII, SalI, and T4DNA ligase were all purchased from TaKaRa (Japan). The primers were synthesized by Guangzhou Sangon Biotech Co., Ltd.

### Cloning and sequencing of the *exsA* gene from *V. alginolyticus* HY9901

A pair of primers *exaA*-F/*exsA*-R were designed based on the *V. alginolyticus* gene sequence (GenBank Number: GU074526.1) (Table 2). PCR was performed according to the previous study of Zhou et al. (18). PCR products were run on 1.5% agarose gels, visualized with ethidium bromide. Then, the recovered PCR products were cloned into the pMD18-T vector, and transformed into *E. coli* DH5α (Table 1). The inserted fragment was sequenced by Sangon Biological Engineering Technology & Services Co., Ltd. (Guangzhou, China). The similarity analyses were based on the method of Pang et al. (22).

**Table 2:** Sequences of primers used in this study.

**Primer name**	**Primer sequence (5^′^-3^′^)**	**Accession number**
ExsA-A1-F	TGAAGATCTTATCTCGCTCCTTGAACAC	This study
ExsA-A1-R	TAGCCACTTGTTTCTACCCTTCATTATTTTGA	
ExsA-A2-F	AGGGTAGAAACAAGTGGCTATCGCGAAATGAA	This study
ExsA-A2-R	AGCGTCGACCAGACGAGAGTTGATGTAGT	
ExsA-F	ATGGATGTGTCAGGCCAACTA	This study
ExsA-R	TCATTTCGCGATAGCCACTTG	
TNF-α-F	TAGAACAACCCAGCAAAC	NC_007130.7
TNF-α-R	ACCAGCGGTAAAGGCAAC	
IL-6-F	GGTCAGACTGAATCGGAGCG	NM_001079833.1
IL-6-R	CAGCCATGTGGCGAACG	
IL-1β-F	TGGACTTCGCAGCACAAAATG	AY340959.1
IL-1β-R	GTTCACTTCACGCTCTTGGATG	
IgM-F	GTTCCTGACCAGTGCAGAGA	AF246193
IgM-R	CCTGATCACCTCCAGCATAA	
TLR5-F	GAAACATTCACCTGGCACA	NC_007131.7
TLR5-R	CTACAACCAGCACCACCAGAATG	
IL-8-F	GTCGCTGCATTGAAACAGAA	XM_001342570.2
IL-8-R	CTTAACCCATGGAGCAGAGG	
IL-6R-F	GCATGTGCTTAAAGTATCCTGGTC	NM_001114318.1
IL-6R-R	TGCAAATTGTGGTCGGTATCTC	
c/ebpβ-F	GCCGTACCAGACTGCTCCGA	NC_007119.7
c/ebpβ-R	AGCCGCTTCTTGCCTTTCCC	
Gata-1-F	GCCTGATAAACTGAATTTAGTA	NC_007122.7
Gata-1-R	GTCGTGCAAAAGAATTATGTGA	
β-actin-F	ATGGATGAGGAAATCGCTGCC	NM_131031.1
β-actin-R	CTCCCTGATGTCTGGGTCGTC	
*hop*-F	CTTCGCTTTCGGTTTGCT	KX245315
*hop*-R	AATACCATCCCACCCTGT	
16S-F	TTGCGAGAGTGAGCGAATCC	NR_044825.2
16S-R	ATGGTGTGACGGGCGGTGTG	
q-CheA-F	CCGCAGTTACCACATCAAGG	This study
q-CheA-R	GCCAGAATCTAAACCAGTCGC	
q-FlaE-F	GTTTGCTTGGTAGTTGCGTTGT	This study
q-FlaE-R	GGCTTAGCGAAAGTGAGTGGA	
q-FlaG-F	AATCGTCACCACATCTCGTCC	This study
q-FlaG-R	AAAGCCGTCAAGAACTGAACAA	
q-FliN-F	GCCGTGAGCAATCAAAGTACC	This study
q-FliN-R	GCGAAGATGAGCGTCGTAAA	
q-FliS-F	TGTCCAAAATTCATCCAACC	This study
q-FliS-R	GGAAAACCTACGCCAGCT	
q-CysN-F	TAATCGGTCGCTTACTCCAT	This study
q-CysN-R	GCAAGGTCGGGCTTTTCA	
q-KdsA-F	ATCGTTGAGAAGTTCGCAGAG	This study
q-KdsA-R	GATGGGTCACGCATTTGTAGT	
q-MinD-F	ACGGCTTGGCTTTCTGGA	This study
q-MinD-R	CGTGACTCTGACCGCATTCT	

### Construction of in-frame deletion mutant of *exsA* gene

The in-frame deletion of *exsA* in the *V. alginolyticus* was generated according to the previous study (23). For the construction of Δ*exsA*, two pairs of primers were firstly designed to obtain the *exsA* upstream homologous arm fragment A (Primers: *exsA*-A1-F and *exsA*-A1-R) and downstream homologous arm fragment B (Primers: *exsA*-A2-F and *exsA*-A2-R). Both fragments contained a 10 bp overlapping sequence and were used as templates for the subsequent PCR procedure, which used primers *exsA*-A1-F and *exsA*-A1-R. The PCR product was ligated into suicide vector pMD4 to generate pMD4-Δ*exsA*. Then, the recombinant suicide plasmid was used to transform into *E. coli* S17-1. The single crossover mutants were acquired by transferring the resulting plasmid directly into *V. alginolyticus* HY9901. Ten percentage sucrose TSA plates were used to select the deletion mutants. HY9901Δ*exsA* was subsequently confirmed by PCR and sequencing using primers *exsA*-F and *exsA*-R.

### Characterization of the Δ*exsA*

The Δ*exsA* phenotype characterized by growth, genetic stability, extracellular protease (ECPase) activity, swarming motility and biofilm Formation. The experimental method of genetic stability was based on previous studies our research group undertook (18). In brief, ΔexsA were seeded into a Tryptic Soya agar (TSA) plate, passed randomly for 30 generations. Its genetic stability was determined by PCR. Growth curves were determined using the method of Wu et al. (24). Extracellular protease activity assay was performed referring to Windle and Kelleher (25). HY9901 and Δ*exsA* were inoculated onto TSA plates coated with sterile cellophane, and then cultured at 28°C for 24 h, washed with PBS, centrifuged at low temperatrue for 30 min, and filtered the supernatant using a 0.22 μm porous membrane. The protease activity of the supernatant was measured at OD_442_ using an azocasein trichloroacetic acid colorimetric assay solution.

Biofilm formation was measured with the method of Zhou et al. (18). Swarming motility was assayed according to the previous method described by Mathew et al. (26). LD_50_ of HY9901 and Δ*exsA* were assessed by the previous study of Chen et al. (27). In brief, a total of 330 fish were blindly separated into three groups. The zebrafish in experimental group were injected with 5 μL HY9901 and Δ*exsA* suspended in PBS containing 10^4^-10^8^ CFU/mL, while negative control group was injected with 5 μL of PBS. Fish were monitored for 14 days or until no morbidities occurred, and LD_50_ of HY9901 and Δ*exsA* was calculated. All experiments were carried out triplicate.

Antibiotic susceptibility testing was performed. Briefly, HY9901 and Δ*exsA* were seeded into TSA plates, and then 30 different antibiotics disks were respectly added into the plates. The plates were incubated for 16 h at 28°C and the diameters of the inhibition zones were measured by Vernier calipers. All experiments were carried out triplicate.

### Transcriptome sequencing

With reference to the method described previously (22), HY9901 and Δ*exsA* cells were cultured in DMEM media at 28°C. The bacterial cells were harvested after 12 h. The series of experiments, including mRNA extraction, RNA fragmentation, cDNA synthesis and RNA-Seq library construction, were conducted by Novogene Co., Ltd. The data generated from the Illumina platform were used for bioinformatics analysis using I-Sanger Cloud Platform (www.i-sanger.com). Besides, the GO terms of the different expression genes were analyzed with the Goatools tool (EMBL, European Molecular Biology Laboratory).

### Validation of transcriptome data

qRT-PCR was used to validate the different expression genes obtained from transcriptome analysis. The related-gene primers sequences were tabulated in Table 2. Besides, the 16 s rRNA gene was used as the internal reference. RNA was extracted, synthetic cDNA and real-time PCR was based on the method of previous study (28).

### *hop* gene expression analysis

#### Detection of HopPmaJ mRNA expression by qRT-PCR after deletion of *exsA* gene

In order to facilitate T3SS secretion, HY9901 and HY9901Δ*exsA* were cultured in DMEM media for 16 h. The primers for *hop* are shown in Table 2. 16S rRNA is used as an internal reference. RNA was extracted, synthetic cDNA and real-time PCR was based on the method of previous study (28).

### β-galactosidase reporter assay

The β-galactosidase reporter assay was carried out as previously described (29). In brief, the recombinant plasmid PME6522 containing the promoter region of *hop* was introduced to HY9901 and HY9901Δ*exsA*, and a LacZ reporter assay was employed to measure the activity and regulation of promoters. The recombinant plasmid PME6522 and related-gene primers are, respectively, shown in Tables 1, 2. The experiments were carried out triplicate.

### Vaccination

The concentration of Δ*exsA* in LD_50_ experiment is 10^5^ cfu/mL, which is not lethal to zebrafish (Table 3). Vaccination was undertaken according to Zhou et al. (18). Briefly, zebrafish were randomly separated into two groups with 80 fish per group. The water temperature was adjusted to 28°C.Fish in the Δ*exsA* group were injected with 5 μL Δ*exsA* bacterial solution (10^5^ cfu/mL), while control fish were injected with 5 μL PBS. The experiment was repeated three times.

**Table 3:** Experiment of LD_50_.

**Concentration (CFU/mL)**	**HY9901**	**Mortality rate (%)**	**Δ*exsA***	**Mortality rate (%)**	**Control (PBS)**	**Mortality rate (%)**
10^8^	10 × 3	90	10 × 3	50	–	
10^7^	10 × 3	80	10 × 3	40	–	
10^6^	10 × 3	60	10 × 3	10	–	
10^5^	10 × 3	20	10 × 3	0	–	
10^4^	10 × 3	20	10 × 3	0	–	
0 (PBS)	–		–		10 × 3	0

### Vaccine safety evaluation

The number of HY9901Δ*exsA* in livers and spleens of selected from three fish in HY9901Δ*exsA* group were investigated to further assess safety of live attenuated HY9901Δ*exsA* vaccine. Briefly, fish were weighed and euthanized to sample liver and spleen once daily for seven consecutive days. All the samples were weighed, homogenized in 1 mL of sterile PBS. The homogenates were diluted serially and spread on TCBS plates, and the plates were incubated at 28°C for 16 h. Colony numbers were counted visually and colonies measuring at least 50 μm were counted. The bacteria counts were calculated by dividing the weights of the tissues and from the mean of three samples. Besides, the identities of the HY9901Δ*exsA* isolates were confirmed by colonial morphology and PCR.

### Immune-related gene of zebrafish expression analysis

Briefly, liver and spleen samples were taken from three fish from each group, respectively, at 1 day before the challenge. The expression levels of immune-related genes were determined by qRT-PCR. Primers are shown in Table 2, and β-actin was used as internal reference. Detailed experimental steps refer to the previous study of Li et al. (28).

### Routine H&E stained sections were analyzed for histopathology

To evaluate the safety of the Δ*exsA* vaccines, livers and spleens of selected from three fish from each group were fixed in neutral buffered 10% formalin, embedded in paraffin and processed for routine histopathological examination. Detailed experimental steps refer to our previous study (30).

### Challenge experiment

Fish were challenged 28 days post vaccination. zebrafish (*n* = 30) were given intramuscular injection of 5 μL 1 × 10^8^ cfu mL^−1^ of *V. alginolyticus* HY9901. The relative percent survival (RPS) of post-challgenged fishes were measured per day in a 14-day time frame. The experiment was repeated three times.

### Statistical analyses

Statistical analyses were conducted using SPSS 17.0 (SPSS Inc., USA). The statistical significance of differences between the wild-type strain and Δ*exsA* mutant, were determined using the Student's *t*-test. Group differences were determined by Duncan's test. Data was considered statistically significant when *p* < 0.05.

### Ethics statement

All animal experiments were conducted strictly based on the recommendations in the “Guide for the Care and Use of Laboratory Animals” set by the National Institutes of Health. The animal protocols were approved by the Animal Ethics Committee of Guangdong Ocean University (Zhanjiang, China).

### Biosecurity

The bacteria protocols were approved by the Biosecurity Committee of Guangdong Ocean University (Zhanjiang, China).

## Results

### Cloning of *exsA* gene and construction of mutant

**Figure 1:**
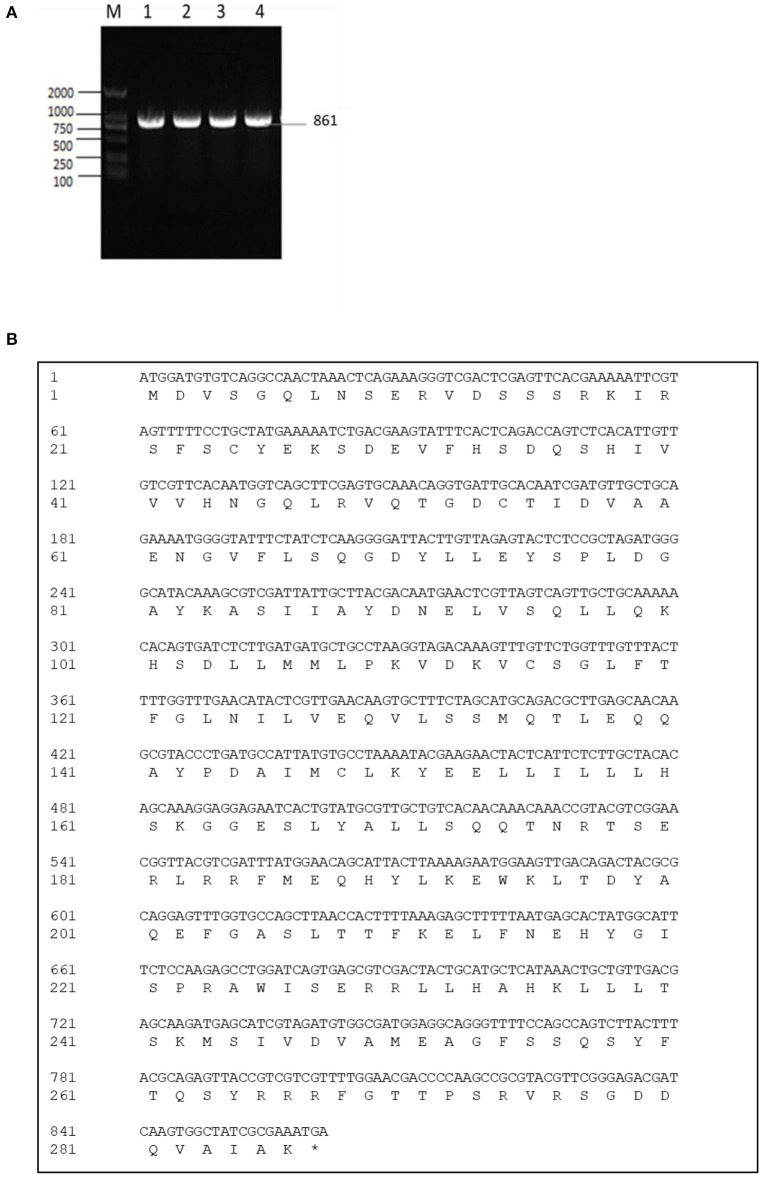
**(A)** Cloning of *exsA* gene. M: DL2000 marker. Lane 1-4: The 861 bp fragment amplified from genomic DNAs of HY9901 using primer pairs of ExsA-F/ExsA-R. **(B)** *exsA* gene nucleotide and its encoded amino acid sequence.

**Figure 2:**
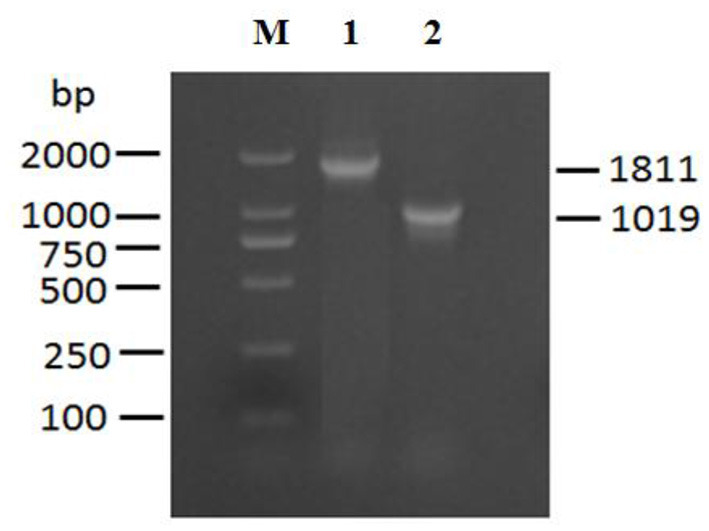
Construction and confirmation of the knockout mutant strain HY9901Δ*exsA*. M: DL2000 marker, Lane 1. A fragment of 1,811 bp is obtained for HY9901 using primer pairs of *exsA*-A1-F/*exsA*-A2-R. Lane 2. A fragment of 11,019 bp is obtained for HY9901Δ*esxA* using primer pairs of *exsA*-A1-F/*exsA*-A2-R.

The exsA gene is 861 bp long, and encodes 286 amino acids (aa) with a predicted molecular weight of 32.549 kDa and a theoretical isoelectric point of 6.0 (*exsA* accession no. MN385414) (Figures 1A,B). To understand the potential function of ExsA in *V. alginolyticus*, an untagged in-frame deletion mutant strain HY9901Δ*exsA* was constructed by Overlap PCR and a dual selection strategy. HY9901 was determined by PCR by generating a fragment of 1,811 bp, while Δ*exsA* was determined by PCR by generating a fragment of 1,019 bp (Figure 2).

### Characterization of the *exsA*

#### HY9901Δ*exsA* was genetically stable

**Figure 3:**
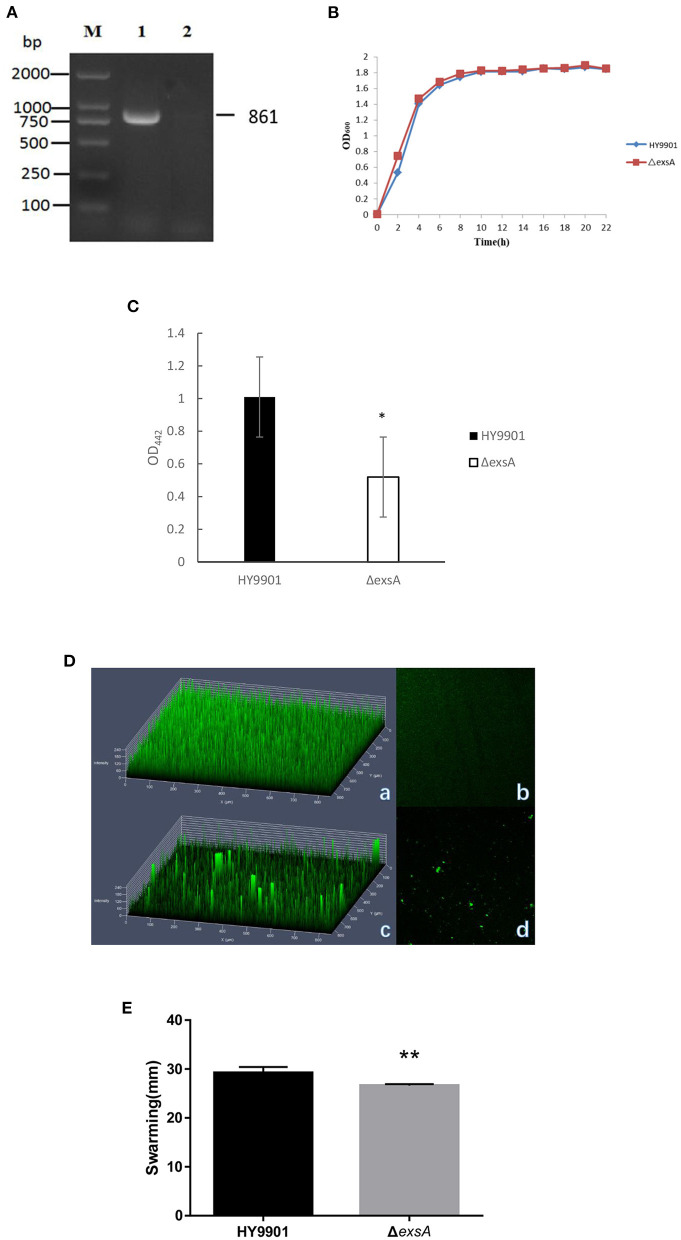
Characteristics of different strains. **(A)** Genetic stability detection of HY9901Δ*exsA* deletion mutantt. M: DL2000 marker; Lane 1. The 861 bp fragment amplified from genomic DNAs of HY9901 using primer pairs of ExsA-F/ExsA-R; Lane 2. The negative result amplified from genomic DNAs of Δ*exsA* using primer pairs of ExsA-F/ExsA-R. **(B)** Growth rates of HY9901Δ*exsA* and wild-type strain HY9901 (wt). Aliquots of cell culture were obtained at various time points and measured for cell density at OD_600_. **(C)** Activity of extracellaluar proteases. The extracellular protease activity was measured at OD_442_. **(D)** Measurement of biofilm by LSCM. (a) HY9901 2.5d diagram. (b)HY9901Δ*exsA* 2.5d diagram. (c) HY9901 2d diagram. (d) HY9901Δ*exsA* 2d diagram; HY9901 Biofilm thickness: 60 ± 10 μm, HY9901Δ*exsA* Biofilm thickness:90 ± 20 μm. (**E)** Swarming diameters were measured after 24 h incubation on TSA containing 0.3% agar plates. **p < 0.01.

After 30 generations of continuous blind transmission of HY9901Δ*exsA*, the *PCR* genome of HY9901Δ*exsA* and wild-type strain HY9901 using primers ExsA-F/ExsA-R, a fragment of 861 bp was obtained for HY9901, while Δ *exsA* is negative. This result indicates that Δ*esxA* has deleted the gene *esxA*, which can stabilize the inheritance (Figure 3A).

#### HY9901 and HY9901Δ*exsA* did not differ for growing

There were no significant differences in growth rates between wild-type strain HY9901 and HY9901Δ*exsA* (*p* > 0.05). The exponential growth phase of the two bacterial strains was from 0 to 4 h, with growth stationary at 16 h, OD_600_ ≈ 1.8 (Figure 3B).

#### The *exsA* is a positive contributor to extracellular protease activity

ECP is a crucial virulence factor, and have a variety of protease activities. After deletion of *exsA* gene, the extracellular protease activity of Δ*exsA* was decreased compared to the wild-type strain HY9901 (*p* < 0.01) (Figure 3C).

#### The biofilm formation ability of HY9901Δ*exsA* decreased impressively

Biofilms are organized groups of microorganisms, usually formed on abiotic or natural surfaces, where organisms are embedded into a matrix composed of extracellular polymers. In comparison to the development observed in HY9901 wild type strain by confocal microscopy, Δ*exsA* showed a decline in biofilm formation capability (*p* < 0.01) (Figure 3D; Table 4).

**Table 4:** Characteristics of different strains.

**Characteristics**	**HY9901**	**Δ*exsA***
Activity of ECPase (A_442_)^a^	1.01 ± 0.2	0.52 ± 0.2^**^
Swarming (mm)^b^	29.2 ± 1.2	26.6 ± 0.3^**^
Biofilm thickness (μm)	60 ± 10	30 ± 5^**^
LD_50_ (cfu/ml)^c^	5.8 × 10^5^	6.3 × 107^**^

** *p* < 0.01.

Values are mean ± standard deviation for three trials.

a Bacteria were incubated in TSB for 18 h at 28°C.

b Swarming diameters were measured after 8 h incubation on TSA containing 0.3% agar plates.

c LD_50_ was evaluated in zebrafish with an average weight of 0.11 ± 0.10 g.

#### The HY9901Δ*exsA* showed an attenuated swarming phenotype

The result of swarming assay was showed in the Table 3. As for swarming activity, the swarming circle of the wild strain HY9901was 29.2 ± 1.2 mm, and Δ*exsA* was 26.6 ± 0.3 mm. The result indicated that the swarming ability of HY9901Δ*exsA* was significantly weakened (Figure 3E; Table 4).

#### LD_50_ determination

In this study, zebrafish were used as models to evaluate the virulence of the HY9901 and Δ*exsA*. The results showed that the 50% lethal dose of Δ*exsA* was 100 times higher than that of wild strain (Tables 3, 4). All of the dead fish exhibited the clinical symptoms of Vibriosis such as ulcers on the skin, hemorrhagic and swelling in the liver and kidney. The results showed that the virulence of HY9901Δ*exsA* was significantly decreased when compared with the HY9901 (*p* < 0.01).

#### Antibiotic susceptibility

The susceptibility of the wild strain HY9901 and Δ*exsA* to 30 antibiotics was determined by disc diffusion method. The result was as follows. The HY9901 was highly resistant to chloramphenicol, while the Δ*exsA* had a zone of inhibition against chloramphenicol. Besides, the Δ*exsA* was more sensitive to kanamycin, minocycline, tetracycline, gentamicin, doxycycline and neomycin (Table 5).

**Table 5:** Drug sensitivity test results of the HY9901 and HY9901Δ*exsA*.

**Antibiotic**	**Dose (μg)**	**Bacteriostatic circle diameter**			
		**HY9901**	**Sensitivity**	**Δ*exsA***	**Sensitivity**
Cefoperazone	75	0.00	R	0.00	R
Oxacillin	1	0.00	R	0.00	R
Clindamycin	2	0.00	R	0.00	R
Ceftazidime	30	0.00	R	0.00	R
Penicillin	10U	0.00	R	0.00	R
Ampicillin	100	0.00	R	0.00	R
Carbenicillin	100	0.00	R	0.00	R
Cefazolin	30	0.00	R	1.00	R
Ceftriaxone	30	0.00	R	0.00	R
Cefradine	30	0.00	R	0.00	R
Piperacillin	100	0.00	R	0.00	R
Cefuroxime	30	0.00	R	0.00	R
SMZ/TMP	23.75/1.25	0.00	R	0.00	R
Mideamycin	30	0.00	R	0.00	R
Vancomycin	30	0.00	R	0.00	R
Cephalexin	30	0.00	R	0.00	R
Polymyxin B	200IU	0.00	R	0.00	R
Norfloxacin	10	0.00	R	0.00	R
Ofloxacin	5	0.00	R	0.00	R
Ciprofloxacin	5	0.00	R	0.00	R
Amikacin	30	11.0	R	11.5	R
Minocycline	30	13.5	I	18.0	S
Tetracycline	30	12.1	R	15.0	I
Gentamicin	10	0.00	R	12.3	I
Furazolidone	300	7.1	R	8.0	R
Chloramphenicol	30	14.5	I	0.00	R
Kanamycin	30	0.0	R	11.0	R
Erythromycin	15	0.0	R	0.0	R
Doxycycline	30	10.5	R	15.0	I
Neomycin	30	0.0	R	11.0	R

*S, susceptible; I, intermediate; R, resistance*.

### Transcriptome sequencing analysis of HY9901 and strain Δ*exsA*

**Figure 4:**
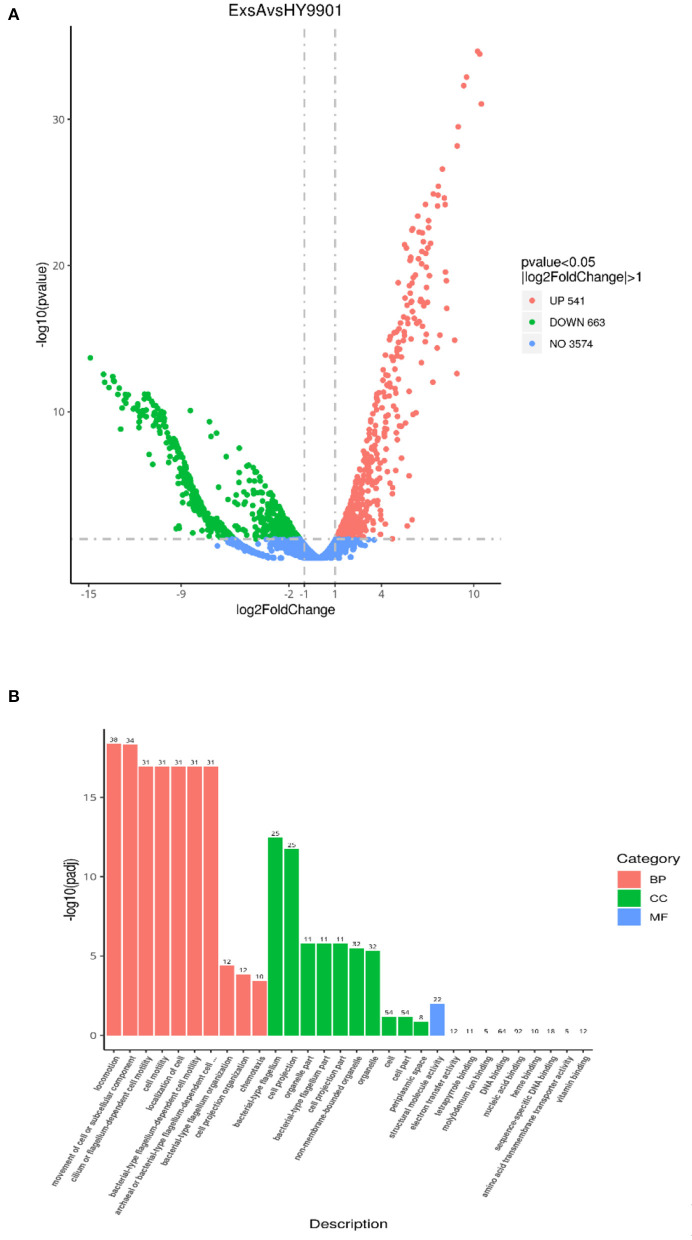
**(A)** Differential analysis volcano map. **(B)** The most enriched GO terms of differentially expressed genes.

The transcriptome sequencing analysis identified a total of 541 upregulated and 663 downregulated differentially expressed genes (DEGs) in Δ*exsA* compared to HY9901 (Figure 4A; Supplementary Table S1). These DEGs were enriched by GO function. Go analysis showed that DEGs of HY9901 and strain Δ*exsA* were related to locomotion, movement of cell or subcellular component and bacterial-type flagellum-dependent cell motility (Figure 4B). We also identified the virulence genes in HY9901 and Δ*exsA* in the transcriptome sequencing data. Compared with HY9901, genes in biofilm formation (*crp, fis, flrA, ompT*), bacterial secretion (*gspC, lip*), two-component system (*epsP, pfeR, phoA*,) and lipopolysaccharide biosynthesis (*lpxK*,) were at lower expression level in Δ*exsA*.

### Validation of transcriptome data at the mRNA level

**Figure 5:**
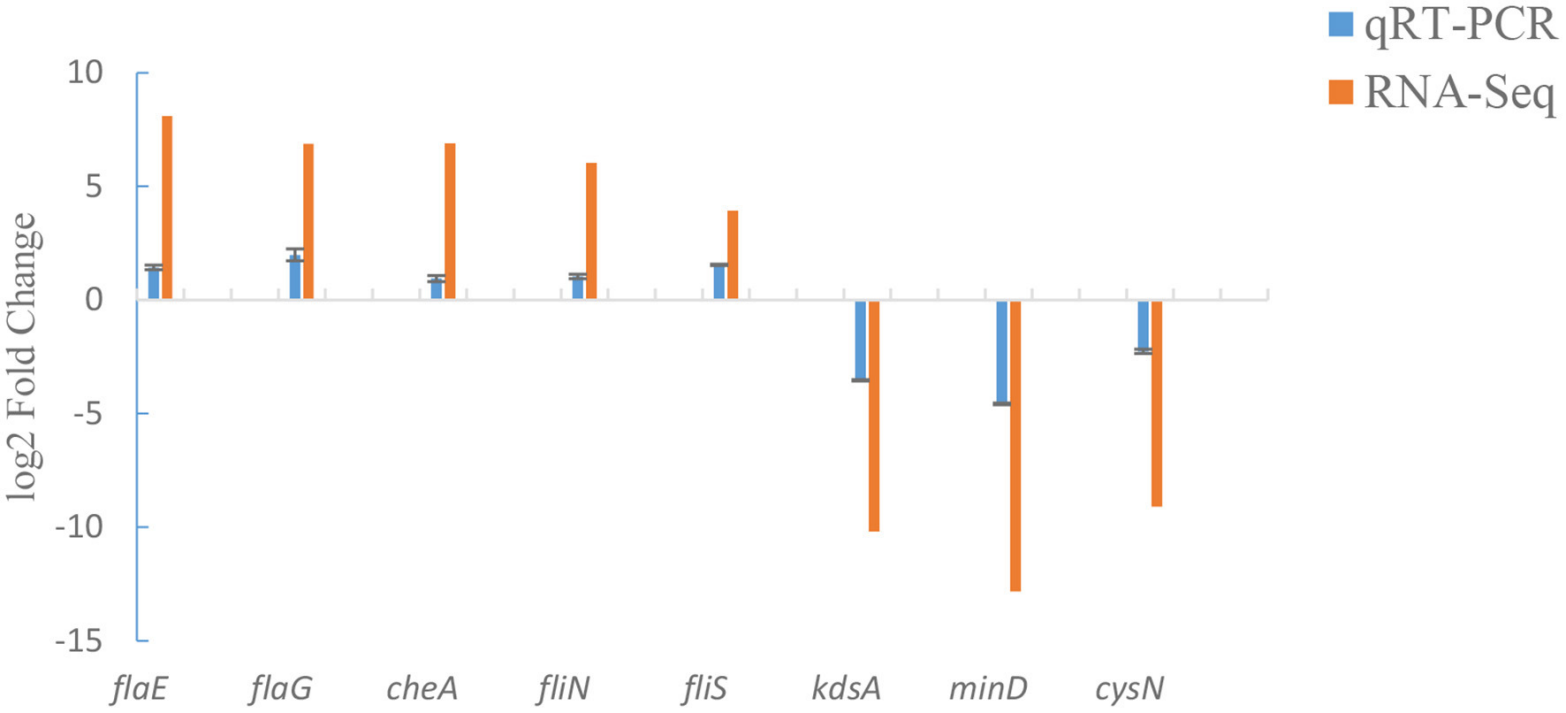
Comparison of qRT-PCR and RNA-Seq.

To verify our transcriptome results, a total of 8 candidate genes (including *flaE, flaG, cheA, fliN, fliS, kdsA, minD* and *cysN*) were analyzed by qRT-PCR. qRT-PCR assay displayed that the changes in mRNA expression of Δ*exsA* were consistent with those of RNA-Seq (Figure 5). These results further validate the reliability of the transcriptome data.

### *hop* gene expression analysis

**Figure 6:**
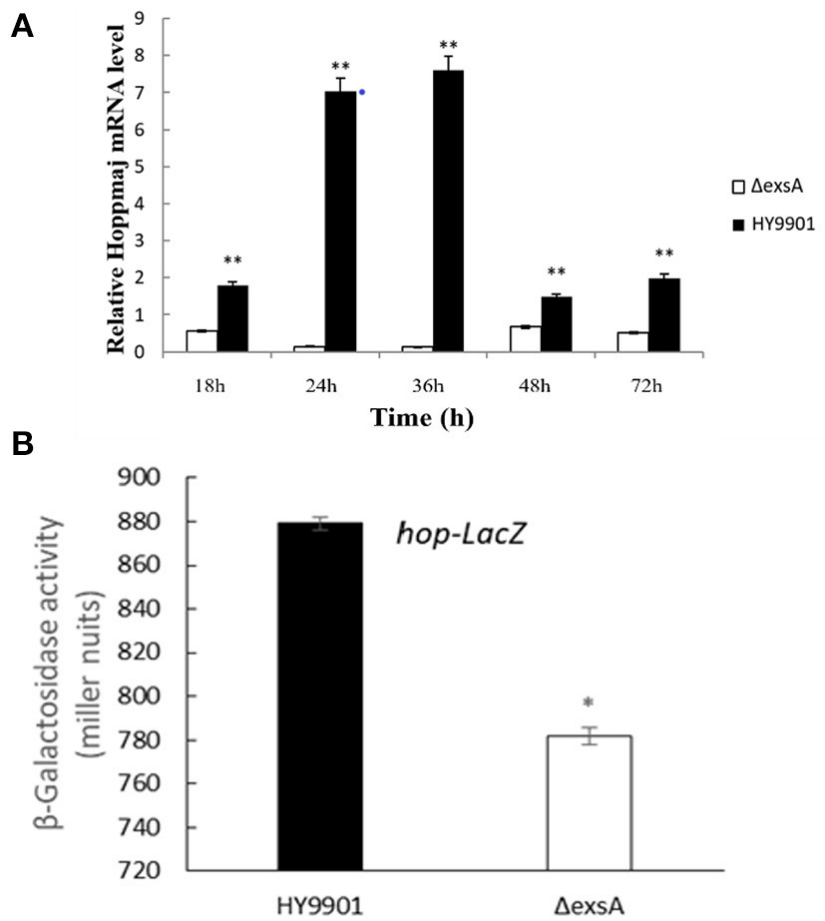
*hop* gene expression analysis. **(A)** Expression of *hop* genes by DMEM between HY9901 wild type and HY9901Δ*exsA*. **(B)** Expression of the LacZ reporter gene between HY9901 wild type and HY9901Δ*exsA*.

The results of qRT-PCR showed that compared with HY9901, Δ*exsA* decreased the expression of *hop* at 18, 24, 36, 48 and 72 h (*p* < 0.01) (Figure 6A). Besides, expression of the LacZ reporter gene was measured with a β-galactosidase enzymatic assay. As shown in Figure 6B, HY9901 wild type strain exhibited high β-galactosidase activity, and Δ*exsA* produced low β-galactosidase activity (*p* < 0.05). These results suggested that *exsA* gene could facilitate the expression of *hop* gene.

### HY9901Δ*exsA* doesn't colonize stably *in vivo*

**Figure 7:**
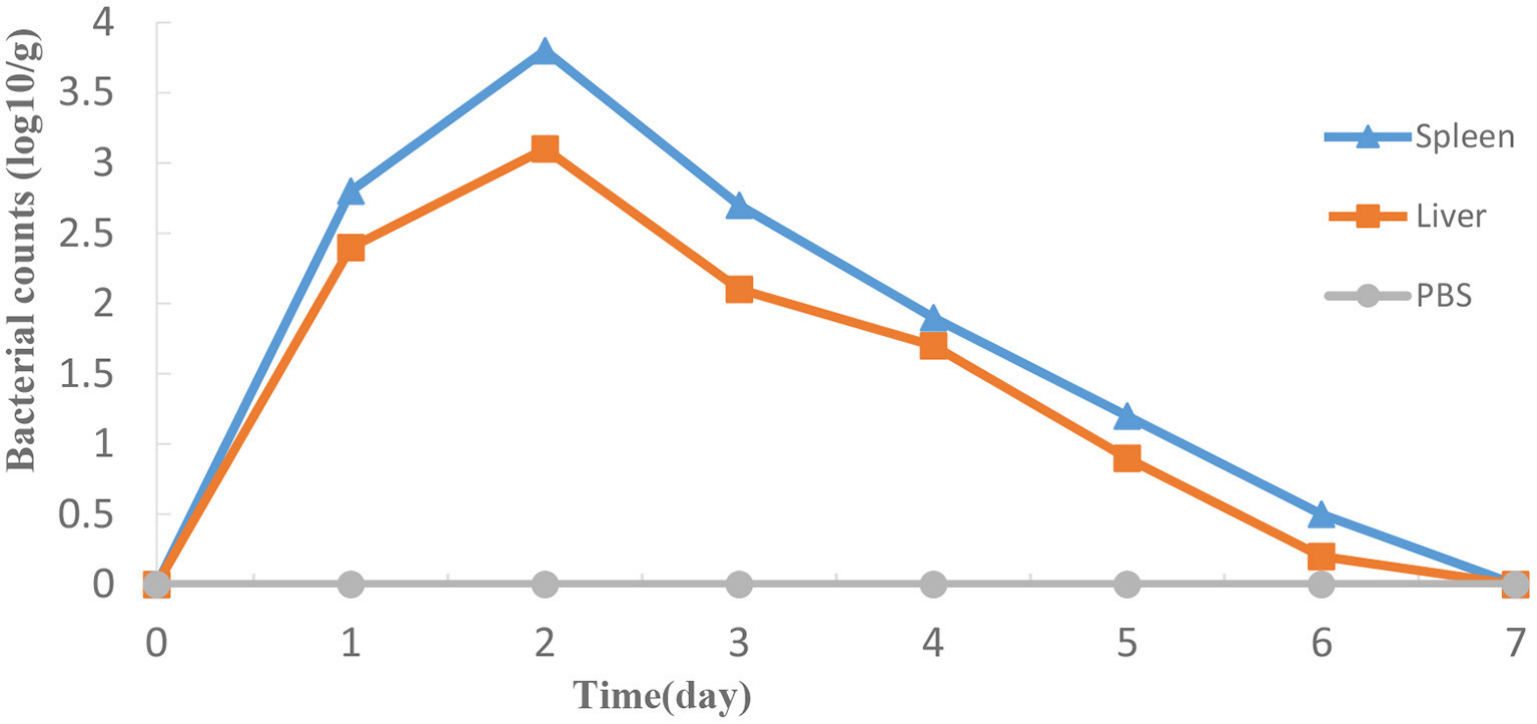
Propagation of HY9901Δ*exsA* in grouper liver **(A)** and spleen **(B)** following i.m. injection with 5 μL 1 × 10^5^ cfu mL^−1^ Δ*exsA*. Control fish were i.m. injection with 5 μL sterile PBS. The number of viable bacteria was shown as the mean ± standard of three samples.

As shown in Figure 7, Δ*exsA* could survive momently in fish liver and spleen then was progressively excreted from the host body. The highest bacterial number was detected in spleen on day 2, followed by the liver.

### HY9901Δ*exsA* has immune protective effects on zebrafish

**Figure 8:**
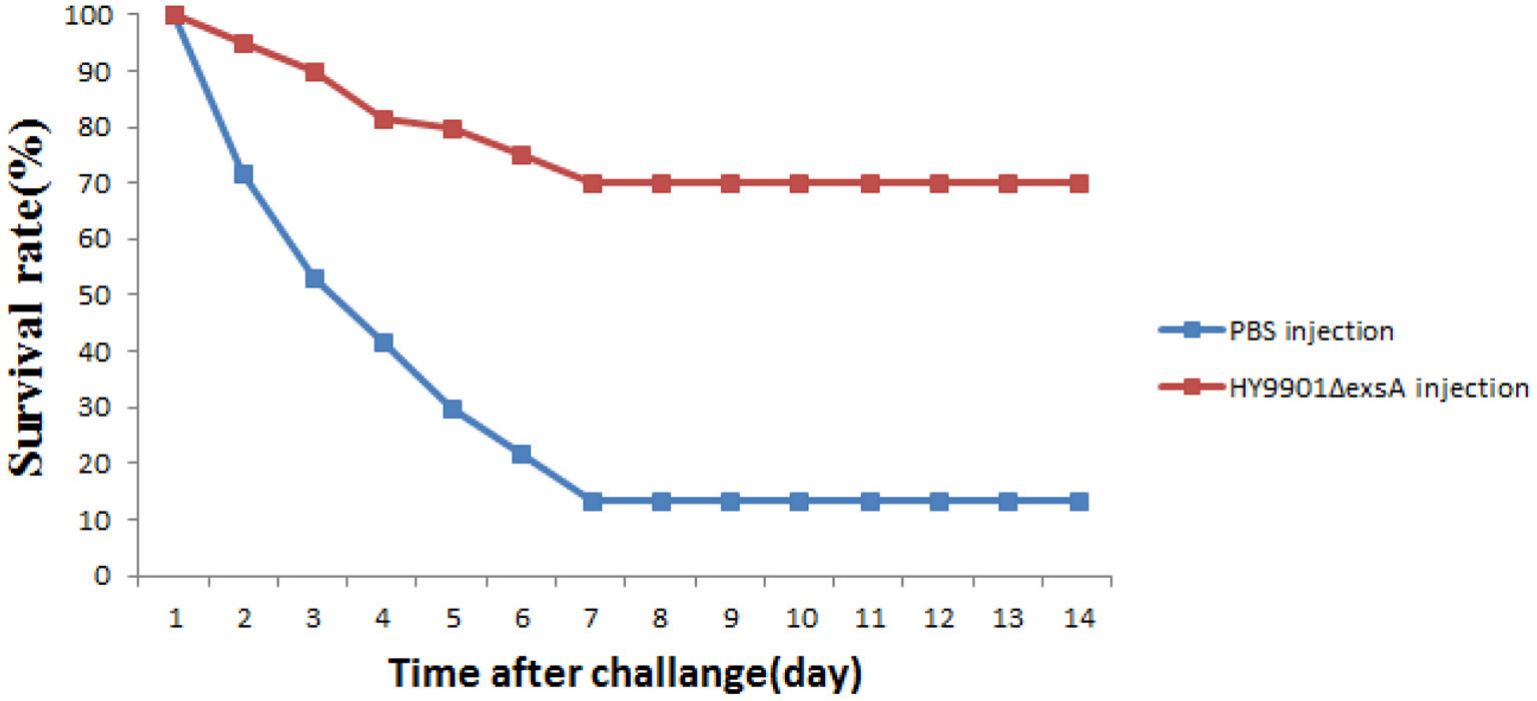
Survival in groups vaccinated with HY9901Δ*exsA* and PBS following challenge with *V. alginolyticus* HY9901.

Zebrafish were vaccinated with PBS and Δ*exsA* by intramuscular injection for 28 days, challenged with the wild type HY9901. As shown in Figure 8, the mortality rate in the control group injected with sterile PBS was 90%, the mortality rate in the injection immunization group was 30%, and the relative percentage survival was 66.7%.

### HY9901Δ*exsA* can upregulate the expression of immune genes in zebrafish after vaccination

**Figure 9:**
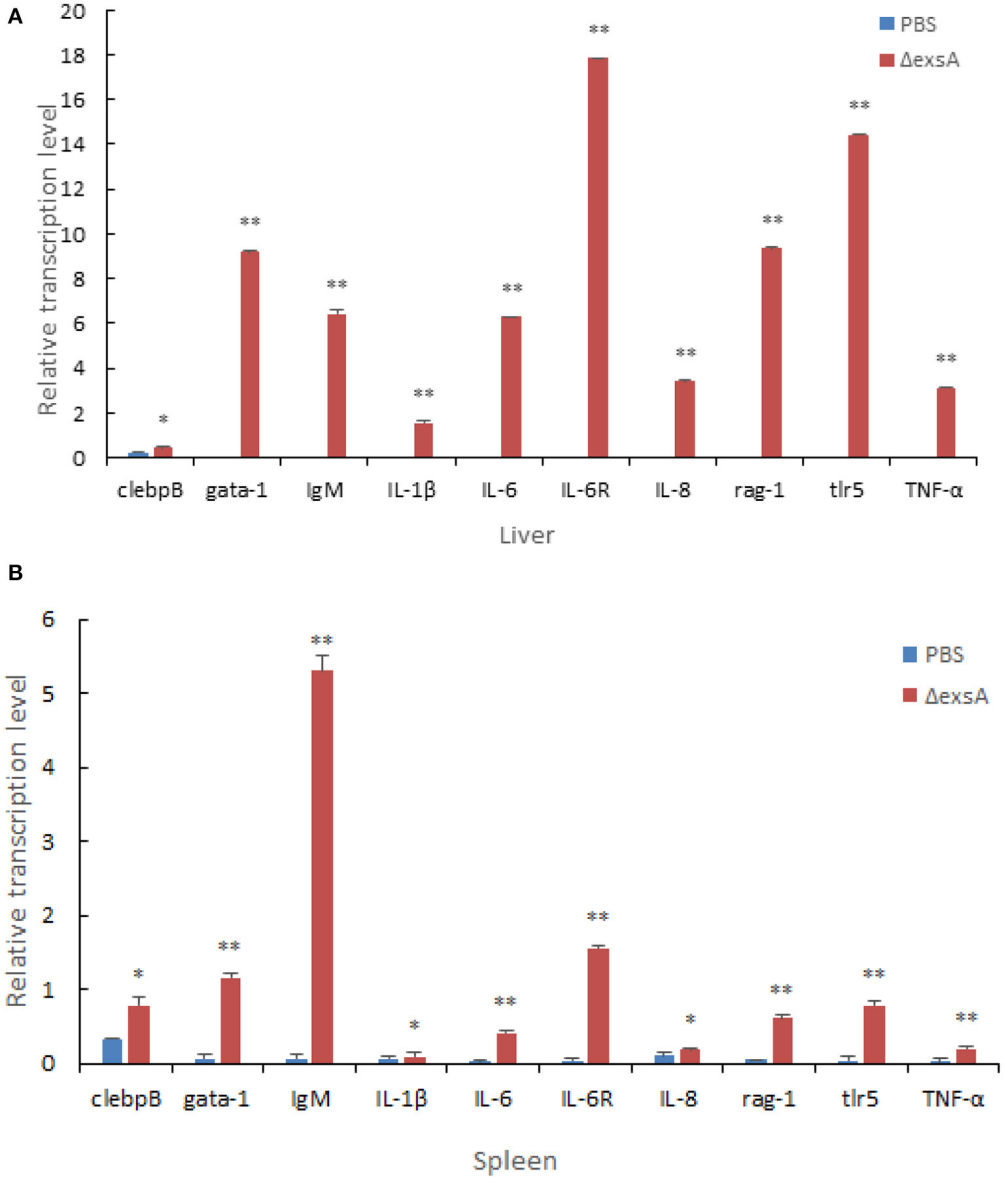
Comparative analysis of the expression of immune-related genes in liver **(A)** and spleen **(B)** of zebrafish given the live attenuated vaccine and unvaccinated zebrafish. The liver and spleen of zebrafish were sampled at 1 day before challenge, and the mRNA level of each immune-related gene was normalized to that of β-actin. Bars represent the mean relative expression of three biological replicates and error bars represent standard deviation.

qRT-PCR was used to analyze the expression of proinflammation and anti-inflammation genes. The results indicated that compared to PBS group, vaccination with Δ*exsA* had significantly increased expression of *IL6, IL8, IL-6R, IL-1*β, *TNF-*α, *tlr5, rag-1, gata-1, IgM*, genes in liver and *gata-1, IL6, IL-6R, IgM, TNF-*α, *rag-1, TNF-*α genes in spleen (*p* < 0.01) (Figure 9).

### The HY9901Δ*exsA* vaccine had a favorable safety profile

**Figure 10:**
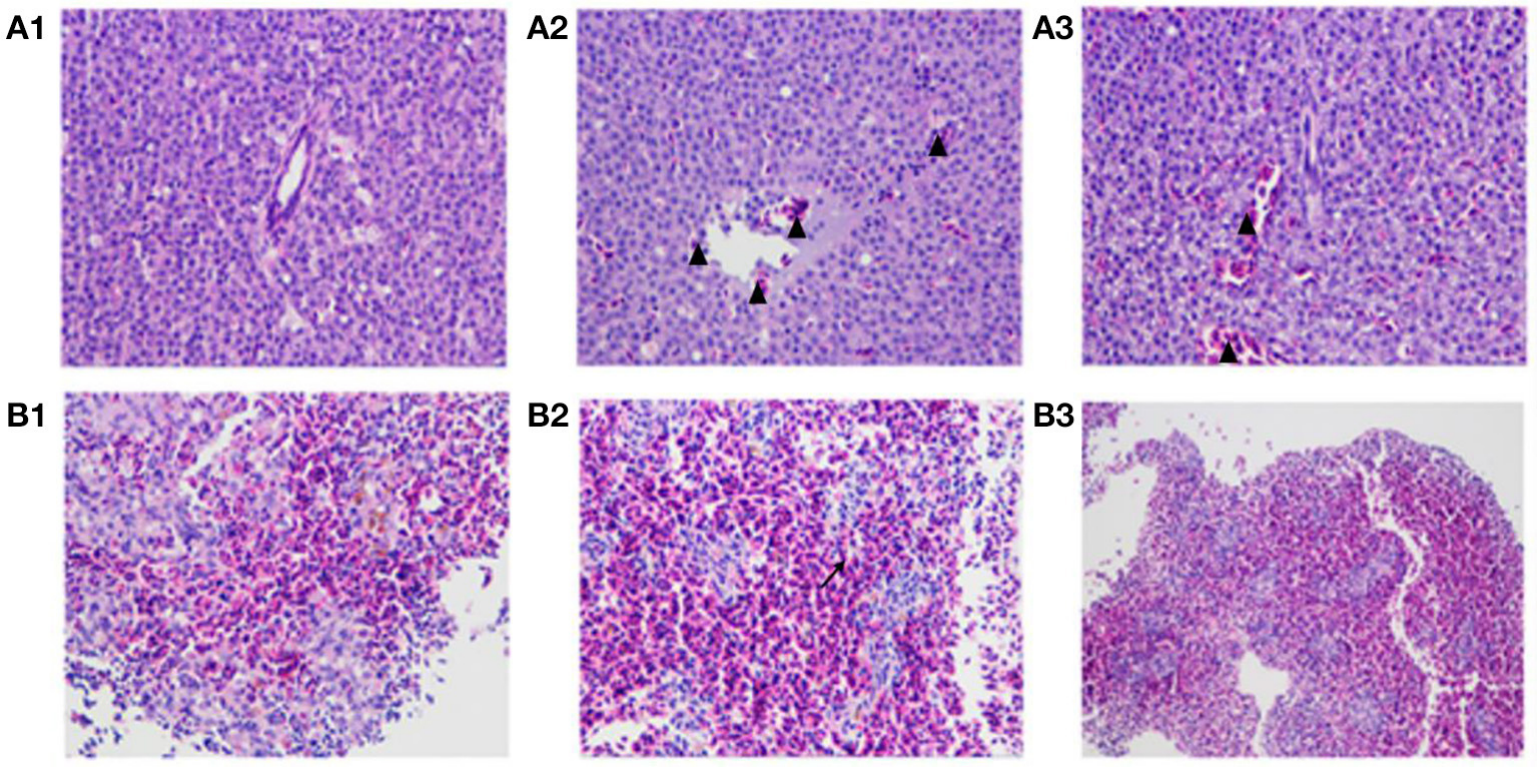
The pathological changes of vaccinated zebrafish. Zebrafish liver tissue section, 400× magnification (**A1**: injected with PBS **A2**: injected with HY9901 **A3**: injected with Δ*exsA*). Zebrafish spleen tissue section, 200 × magnification (**B1**: injected with PBS **B2**: injected with HY9901 **B3**: injected with Δ*exsA*). The triangle (▴) represents hyperemia, and the arrow (**↗**) represents the blurred boundary of lymphocytes in the figure.

As shown in Figure 10, pathological features, such as liver congestion and the boundaries between the lymphocytes in the spleen, were observed visually in the tissue sections of the zebrafish injected wild-type strain HY9901. There was a little number of bleeding spots and congestion in the zebrafish immunized by Δ*exsA* for 28 days, but not in PBS group.

## Discussion

In recent years, vibriosis outbreaks in China have caused serious economic losses, and overuse of antibiotics has driven the emergence and spread of resistance. In this context, the development of a vibrio-based vaccine might be a valuable alternative. Live attenuated vaccines closely mimic natural infection and, *via* specific antibody and cell-mediated immune responses, usually provide excellent protection against infection (31). Recently, attenuated virus strains can be constructed by knockout of the virulence genes. In this study, we knocked out the T3SS gene *exsA* of *V. alginolyticus*, probed into its biology and pathogenicity, and evaluated its effect as a live attenuated vaccine. And the genetic stability result indicated that the genetic information of the Δ*exsA* was stably inherited to offspring after 30 generations.

Biofilms are an important protective mechanism in which microbes form a strong shield against several antimicrobials (32). Vibrio polysaccharide (VPS) is critical for the formation of biofilm architecture in *Vibrio cholera* (33, 34). Flagella-mediated motility is required for the initial stages of biofilm formation (35), and deletion of *V. parahaemolyticus* polar flagellum genes leads to defective biofilm formation (36). Although there was no significant difference in growth between HY9901 and Δ*exsA*, the biofilm formation ability test showed that the mutant strain Δ*exsA* was significantly decreased compared to the wild-type strain HY9901. It is tempting *to* speculate that *exsA* may represses the expression of flagellum-related genes (*flaA* and *fliG*) or genes related to biofilm matrix polysaccharides, resulted in reduced ability to form mature biofilms. Extracellular products (ECP) are mainly thought to be characteristics of the virulent strain of *V. alginolyticus* (20). In this study, the extracellular protease activity of Δ*exsA* was decreased compared to the wild-type strain HY9901, which suggest *exsA* gene may be a positive contributor to activity of ECP in *V. alginolyticus*. ExsA may promote related gene expression of ECP such as *vhh* and *ptlh*, but this needs further investigation. Flagellar motility has long been recognized as a major colonization and virulence factor in many bacterial pathogens, including *Pseudomonas aeruginosa* (37) and *Vibrio cholera* (38). The flagella contribute to swarming motility and enable bacteria to invade into host cells to maintain their ecological niches *in vivo* (39). In this study, Δ*exsA* had suppressed swarming motility, which indicated that *exsA* was a positive contributor to swarming motility in *V. alginolyticus*. We could speculate that *exsA* might play an important role in regulating the expression level of flagella *via* an unclear mechanism. However, the regulatory mechanism is still unknown and also needs further investigation. Compared to HY9901, the LD_50_ of Δ*exsA* was decreased by 20 orders. From these results, we can find that *exsA* is related to the pathogenesis of *V. alginolyticus*.

The origin and molecular basis of bacterial resistance is the presence of antibiotic resistance genes (ARGs). An organism can acquire antibiotic resistance through mutation, horizontal gene transfer, or inheriting resistance genes from other organisms (40, 41). Besides, it is also possible to acquire antimicrobial resistance by mutation, for example changing the antimicrobial target site (42). *V. alginolyticus* has been reported to be resistant to ampicillin, vancomycin, and cephalothin (43). The results of Raissy et al. (44) showed that *strB, tetS*, and *ermB* genes, which encode streptomycin, tetracycline and erythromycin, respectively, could be detected in several strains of *V. alginolyticus* from seafood in Persian Gulf. Further, the author found that some strain not containing *tesT* gene was resistant to tetracycline and induced that there were other genes causing tetracycline resistance (44). In this study, HY9901 was highly resistant to chloramphenicol, while Δ*exsA* was sensitive. Besides, the Δ*exsA* was more sensitive to kanamycin, minocycline, tetracycline, gentamicin, doxycycline and neomycin. It could be guessed that the *exsA* gene might be related to drug resistance genes and regulate the expression of drug resistance-related genes. Nevertheless, it needs to be further studied.

The transcriptome is a collection of all RNA molecules in a cell, which reflects the expression status of the entire genome. It is essential for deciphering the functional complexity of the genome and to obtain a better understanding of cellular activities in organisms, including growth, development, disease, and immune defense (45–47). In the present study, a total of 541 upregulated and 663 downregulated differentially expressed genes in Δ*exsA* compared to HY9901. The outcome of GO analysis revealed that these genes were mainly enriched in a number of GO terms, including locomotion process, movement of cell or subcellular component process, non-membrane-bounded organelle process, organelle process, cilium or flagellum-dependent cell motility process and cell motility process. These results further suggest that exsA gene can regulate the expression of flagella related genes to affect the virulence of *V. alginolyticus*. Nonetheless, its specific regulatory mechanism needs further investigation.

The T3SS is a membrane-embedded nanomachine and it can deliver T3SEs from pathogen to host. The T3SS secretion *in vitro* and *in vivo* is complex, and its pathway is modulated by a single regulatory protein or several interacting regulatory proteins. For example, In *Pseudomonas savastanoi* HrpR and HrpS form a hetero-hexamer, which activates the expression of HrpL, inducing all T3SS genes by binding to a “hrp box” in promoters (48). ExsA is an activator of transcription in T3SS. A previous study indicates that in *P. aeruginosa impA* gene encoding an extracellular metalloprotease is under the regulation of ExsA, and ExsA can directly regulate the transcription of *impA* by binding to its upstream region, demonstrated in electrophoretic mobility shift assay (EMSA) (49). The results of Liu et al. (21) indicated that in *V. alginolyticus* ExsA was a positive regulator in effector proteins Va1686 and Va1687. HopPmaJ is one of the T3SEs from *V. alginolyticus*, which has been reported to attack the host efficiently (50). In this study, the deletion of *exsA* is responsible for the down-regulation of *hop* gene for various time periods, which was demonstrated in qRT-PCR assay. Furthermore, we also found that *exsA* gene could facilitate the expression of *hop* gene as well as acting as positive regulators. From these results, we can draw a conclusion that *exsA* can promote the expression of *hop* gene by binding the promoter regions, which may facilitate HopPmaJ protein secretion.

As a model of good vertebrate animals, zebrafish have been widely used for the study of immunity from a unique perspective. The low cost, rapid development and high fecundity of zebrafish makes it ideal as a vaccine-screening tool (24). It is reported that the *V. parahaemolyticus* can infect zebrafish larvae and activate the innate immune response (51). A previous study indicated that Th17 cells were activated following vaccination of zebrafish (52). Therefore, it is achievable to use zebrafish to investigate the effectiveness of a Vibrio vaccine. In the present study, the RPS of zebrafish vaccinated with Δ*exsA* reached 77.6%, and it was significantly higher than that of the control group.

TLR5 can activate the innate immune system by recognition of bacterial flagellin (53). *IgM* of fish plays an important role in resisting diseases, similar function as *IgM* in mammals (54). *IgM* plays a vital role in immunity, driving direct antimicrobial functions including complement activation, opsonophagocytosis, and agglutination. *IL-1*β and *IL-6* are important cytokines involved in regulating inflammatory response (55, 56). *TNF-*α and *IL-8* are important inflammatory cytokines that mediate the body inflammatory responses and have important roles in the physiological functions and pathological processes (57). In the present study, the elevated expression of immune-related genes (*IgM, IL-1*β, *IL-6*, and *TNF-*α), confirmed that Δ*exsA* can effectively induce the protective immune response of zebrafish associated with proinflammatory and immunoglobulin activity. Tissue sections results indicated a favorable safety profile of Δ*exsA* vaccines. In summary, these results showed that the Δ*exsA* could provide protection against *V. alginolyticus* and has the potential as an attenuated live vaccine. In addition to antibiotics, vaccines are one of the most important means to prevent and control of *V. alginolyticus*, which has great significance for aquaculture industries.

## Conclusions

Taken together, we have successfully constructed an in-frame deletion strain of Δ*exsA* and investigated its physiology and pathogenicity. We found that Δ*exsA* could decrease ECPase activity and swarming motility, and the *exsA* gene regulates *hop* genes expression. Moreover, Δ*exsA* exhibited a high level of protection against *V. alginolyticus* challenge, and could induce protective immune responses in zebrafish. These results may provide experimental evidence for the importance of *exsA* in *V. alginolyticus* and provide a reference for further study into this virulence and infection mechanism.

## Data availability statement

The datasets presented in this study can be found in online repositories. The names of the repository/repositories and accession number(s) can be found in the article/Supplementary material.

## Ethics statement

The animal study was reviewed and approved by the Animal Ethics Committee of Guangdong Ocean University (Zhanjiang, China).

## Author contributions

WZ and HP designed the experiment. FZ, HF, LC, and XX generated experimental data. WZ and JW wrote manuscripts. HP, JJ, and NW conceived the work and critically reviewed the manuscript. WZ, LC, HF, JW, FZ, XX, JJ, NW, and HP have made extensive contributions to the work in this manuscript. All authors contributed to the article and approved the submitted version.

## Funding

This work was supported by the National Natural Science Foundation of China (No. 32073015), Natural Science Foundation of Guangdong Province (No. 2021A1515011078), Undergraduate Innovation Team of Guangdong Ocean University (No. CCTD201802), Innovation and Entrepreneurship Training Program for College Students (No. CXXL2022005), Science and Technology Innovation Cultivation Program for College Student (No. pdjh2021b0239), and Guangdong Postgraduate Education Innovation Project.

## Conflict of interest

The authors declare that the research was conducted in the absence of any commercial or financial relationships that could be construed as a potential conflict of interest.

## Publisher's note

All claims expressed in this article are solely those of the authors and do not necessarily represent those of their affiliated organizations, or those of the publisher, the editors and the reviewers. Any product that may be evaluated in this article, or claim that may be made by its manufacturer, is not guaranteed or endorsed by the publisher.
